# Oral Microbiome Alterations and SARS-CoV-2 Saliva Viral Load in Patients with COVID-19

**DOI:** 10.1128/Spectrum.00055-21

**Published:** 2021-10-13

**Authors:** Emily Happy Miller, Medini K. Annavajhala, Alexander M. Chong, Heekuk Park, Yael R. Nobel, Ali Soroush, John W. Blackett, Anna Krigel, Meaghan M. Phipps, Daniel E. Freedberg, Jason Zucker, Ellen D. Sano, Anne-Catrin Uhlemann, Julian A. Abrams

**Affiliations:** a Department of Medicine, Columbia University Irving Medical Center, New York, New York, USA; b Microbiome and Pathogen Genomics Collaborative Center, Department of Medicine, Columbia University Irving Medical Center, New York, New York, USA; c Department of Emergency Medicine, Columbia University Irving Medical Center, New York, New York, USA; University of Illinois at Urbana Champaign

**Keywords:** COVID-19, SARS-CoV-2, saliva microbiome, viral load

## Abstract

Bacterial-viral interactions in saliva have been associated with morbidity and mortality for respiratory viruses such as influenza and SARS-CoV. However, such transkingdom relationships during SARS-CoV-2 infection are currently unknown. Here, we aimed to elucidate the relationship between saliva microbiota and SARS-CoV-2 in a cohort of newly hospitalized COVID-19 patients and controls. We used 16S rRNA sequencing to compare microbiome diversity and taxonomic composition between COVID-19 patients (*n* = 53) and controls (*n* = 59) and based on saliva SARS-CoV-2 viral load as measured using reverse transcription PCR (RT-PCR). The saliva microbiome did not differ markedly between COVID-19 patients and controls. However, we identified significant differential abundance of numerous taxa based on saliva SARS-CoV-2 viral load, including multiple species within *Streptococcus* and *Prevotella*.

**IMPORTANCE** Alterations to the saliva microbiome based on SARS-CoV-2 viral load indicate potential biologically relevant bacterial-viral relationships which may affect clinical outcomes in COVID-19 disease.

## INTRODUCTION

Biological factors that influence SARS-CoV-2 acquisition and subsequent disease severity are not well understood, particularly with respect to potential interactions between the virus and the human microbiome. Given the extensively reported detection of SARS-CoV-2 in saliva and the possibility of transmission via saliva (1, 2), the oral microbiome may represent one such key constituent and viral reservoir. Oral dysbiosis has been linked to many local and systemic diseases, including periodontitis, and could potentially influence COVID-19 disease severity (3–5). Defining the oral microbiome in COVID-19 disease is a necessary step in determining if factors such as oral hygiene could be a modifiable risk factor for severe disease (6).

It has been suggested that oral and respiratory tract microbiota could similarly express enzymes such as the transmembrane serine protease 2 (TMPRSS2), which may enhance viral entry into host cells and further viral infection (7). Results of a recent study of bronchoalveolar lavage fluid from patients with and without COVID-19 suggest that components of the host microbiome can modify heparan sulfate, a key cofactor to SARS-CoV-2 infectivity (8). Direct interaction between influenza and components of the oral microbiome, such as neuraminidase-producing streptococci, is thought to result in increased viral load (9). These studies support the hypothesis that the host microbiome is an important mediator of disease severity in respiratory viral infections. However, the relationship between oral microbiota and SARS-CoV-2 is currently poorly defined.

Here, we characterize the saliva microbiome and saliva SARS-CoV-2 viral load in COVID-19 and control patients hospitalized during the peak of the COVID-19 outbreak in New York City. Bacterial composition did not differ markedly between COVID-19 patients and controls. However, we identified numerous differentially abundant bacterial taxa associated with SARS-CoV-2 saliva viral load, providing evidence for bacterial-viral interactions in the saliva and suggesting that the host microbiome may represent a cofactor affecting disease course.

## RESULTS

### Clinical characteristics of patient cohort.

We collected saliva within 24 h of hospitalization from 53 COVID-19 patients who tested positive for SARS-CoV-2 based on nasopharyngeal swabs and who did not require intensive care at admission. During the study period, SARS-CoV-2 testing was performed on all patients admitted to the hospital, and we also enrolled 59 control patients who were newly hospitalized and confirmed to be SARS-CoV-2 negative (Table S2). Demographics of case and control cohorts were largely comparable, with no significant differences in age, sex, race, or ethnicity (Table 1). However, SARS-CoV-2 positive patients did have significantly higher median body mass index (BMI; 29.5 versus 26.4, *P* = 0.04). Notably, in our cohort, control patients had a significantly higher median Charlson comorbidity index (3 versus 2, *P* = 0.02), driven by higher rates of coronary artery disease (CAD; 23.7% versus 5.7%, *P* = 0.02) and chronic kidney disease (CKD; 27.1% versus 11.3%, *P* = 0.06). Compared to controls, COVID-19 patients had similar rates of receipt of antibiotics within 48 h of admission (*P* = 0.62) but were more likely to receive supplemental oxygen (*P* = 0.03), primarily via nasal cannula (30.2% of COVID-19 patients). One patient received remdesivir prior to saliva sample collection. With regard to hospital course and outcomes, relatively few COVID-19 patients had clinical decompensation (*n* = 6, 11.3%) or died in the hospital (*n* = 4, 7.5%).

**TABLE 1:** Demographic and clinical characteristics of newly hospitalized COVID-19 and non-COVID-19 patients^*a*^

Characteristic	Value for non-COVID-19^*b*^ (*n* = 59)	Value for COVID-19^*b*^ (*n* = 53)	*P* value^*c*^
Age (mean [SD])	56.2 (16.8)	56.5 (16.1)	0.93
Male (%)	36 (61.0)	24 (45.3)	0.14
Race (%)			0.49
Black	16 (27.1)	20 (37.7)	
White	14 (23.7)	11 (20.8)	
Other/unreported	29 (49.2)	22 (41.5)	
Ethnicity (%)			0.88
Hispanic/Latinx	26 (44.1)	24 (45.3)	
Not Hispanic/Latinx	22 (37.3)	21 (39.6)	
Not specified	11 (18.6)	8 (15.1)	
BMI (median [IQR])	26.4 (23.2, 30.3)	29.5 (24.7, 35.8)	0.04^*c*^
HTN (%)	35 (59.3)	29 (54.7)	0.76
DM (%)	25 (42.4)	19 (35.8)	0.61
CKD (%)	16 (27.1)	6 (11.3)	0.06
Underlying kidney disease (%)	17 (28.8)	8 (15.1)	0.13
Any pulmonary disease (%)	14 (23.7)	12 (22.6)	1.00
Any liver disease (%)	5 (8.5)	3 (5.7)	0.72^*e*^
CAD (%)	14 (23.7)	3 (5.7)	0.02
Charlson comorbidity index (median [IQR])	3 (2, 5.5)	2 (1, 4)	0.01^*d*^
Oxygen rank severity (%)			0.03^*e*^
No supplemental oxygen	49 (83.1)	32 (60.4)	
Nasal cannula	8 (13.6)	16 (30.2)	
Noninvasive ventilation	2 (3.4)	5 (9.4)	
WBC (median [IQR])^*f*^	8.99 (7.08, 11.89)	7.01 (5.37, 10.28)	<0.01
NLR (median [IQR])^*f*^	4.20 (2.35, 8.29)	3.84 (2.57, 7.52)	0.65
Antibiotics ≤48 h prior to saliva collection (%)^*g*^	24 (40.7)	25 (47.2)	0.62
Composite outcome (%)			0.68
Deceased	3 (5.1)	4 (7.5)	
Decompensated (no death)	1 (1.7)	2 (3.8)	
Discharged (no death)	55 (93.2)	47 (88.7)	

a BMI, body mass index; HTN, hypertension; DM, diabetes mellitus; CKD, chronic kidney disease; CAD, coronary artery disease; WBC, white blood cell count; NLR, neutrophil-lymphocyte ratio.

b As determined via clinical testing of nasopharyngeal swabs using RT-PCR.

c Categorical variables were compared using the chi-squared test, unless indicated as below. Continuous variables were assessed for normal or normal-like distribution and compared using *t*-tests, unless indicated as below.

d Kruskal-Wallis test was used due to nonnormal distribution.

e Fisher’s exact test was used due to at least one expected value of ≤5.

f Lab values as measured upon admission; not available for all patients (WBC *n* = 58/59 of controls, 53/53 of cases; NLR *n* = 56/59 of controls, 47/53 of cases).

g Inpatient antibiotic use, as determined through chart review.

### Saliva microbiome in COVID-19 patients and controls.

**FIG 1:**
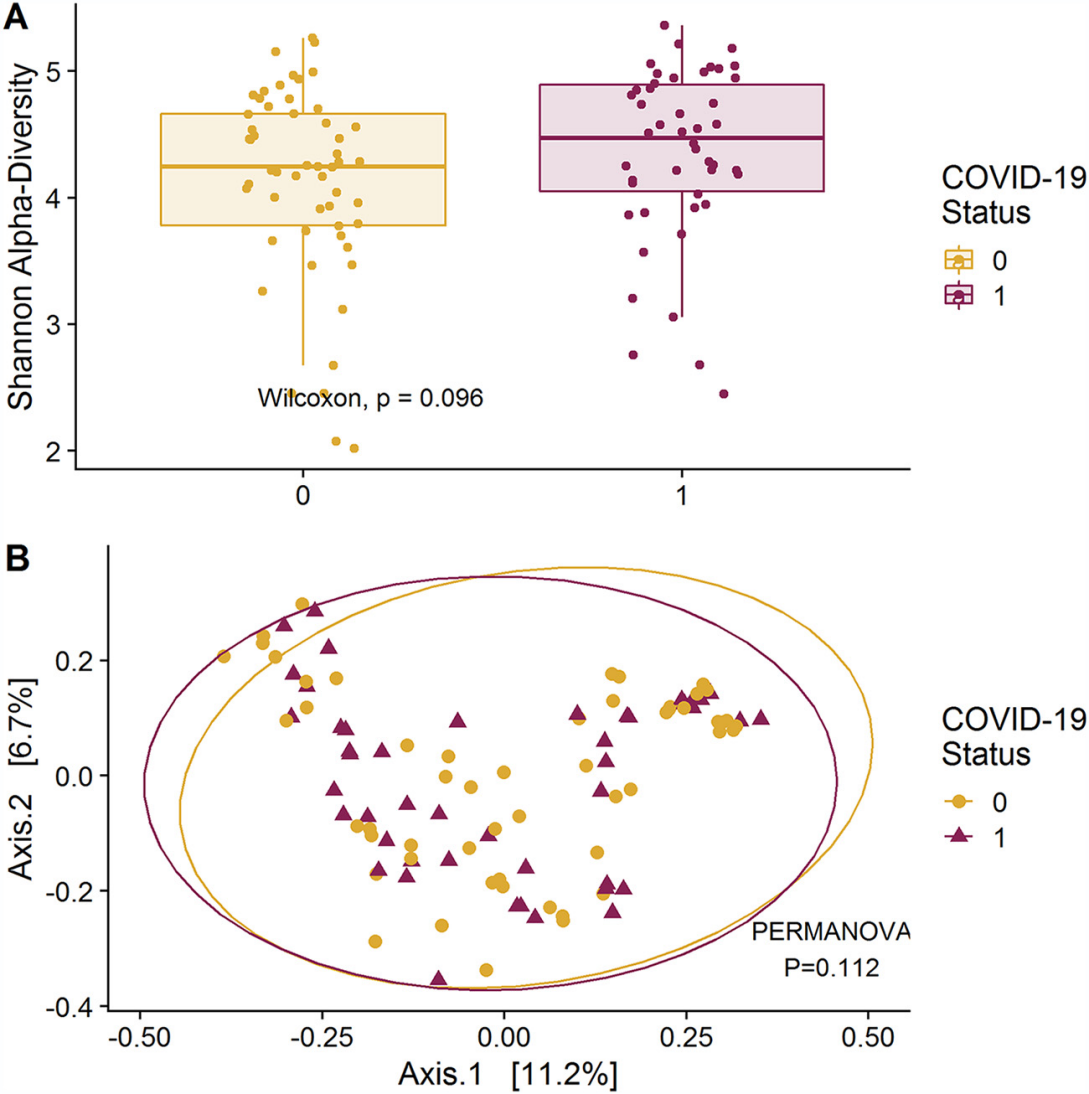
Saliva microbiome diversity in patients with versus without COVID-19. (A) Shannon α-diversity and (B) UniFrac β-diversity in saliva collected from patients with SARS-CoV-2-positive versus -negative nasopharyngeal swabs via clinical testing upon admission. Kruskal-Wallis test was used to compare Shannon α-diversity, and permutational ANOVA (PERMANOVA) was used to compare UniFrac β-diversity. Normal data ellipses for each group are shown over the β-diversity plot in panel B.

Of the enrolled patients, we were able to perform saliva microbiome analyses by 16S rRNA gene sequencing on 46 COVID-19 patients (86.8%) and 54 controls (91.5%). There were no significant differences in α-diversity comparing COVID-19 patients and controls (Shannon *P* = 0.10; Chao *P* = 0.21), and there was no evidence of clustering by COVID-19 status in β-diversity analyses (permutational multivariate analysis of variance [PERMANOVA] *P* = 0.11) (Fig. 1; Fig. S1). However, differential abundance analyses did show significant alterations in three amplicon sequence variants (ASVs), including enrichment of Prevotella pallens in COVID-19 patients and enrichment of Rothia mucilaginosa and a Streptococcus spp. in control patients (DESeq2, adjusted *P* value [*P*_adj_] of <0.05).

### Saliva SARS-CoV-2 viral load in COVID-19 patients.

Cycle threshold (*C_T_*) values from SARS-CoV-2 reverse transcription (RT-PCR) testing can be used as a surrogate for viral load and have been associated with patient outcomes (10). We performed RT-PCR on saliva from both COVID-19 and control patients. Among COVID-19 patients, 28 (62%) had detectable SARS-CoV-2 in the saliva, and one patient who tested negative for SARS-CoV-2 on nasopharyngeal swab had detectable viral load in their saliva. Thus, RT-PCR testing of saliva in this cohort had 65.1% (95% confidence interval [CI] 49.1% to 79.0%) sensitivity and 98.1% (95% CI 90.1% to 99.9%) specificity to identify COVID-19 patients (with nasopharyngeal swab results as the gold standard). Detection of SARS-CoV-2 in saliva was not significantly associated with clinical characteristics among COVID-19 patients.

We then categorized COVID-19 patients based on saliva viral load as follows: negative, low viral load (*C_T_* of ≥30.4 [the median value among COVID-19 patients with detectable SARS-CoV-2 in saliva] and <40), and high viral load (*C_T_* < 30.4). Across these groups, we did not observe any significant differences in demographic or clinical characteristics (Table S3). There was no association between saliva viral load and symptom duration prior to hospital admission. Saliva viral load was not associated with clinical decompensation or death, although these outcomes occurred in relatively few patients.

### Differences in saliva microbiome composition based on saliva SARS-CoV-2 viral load in COVID-19 patients.

**FIG 2:**
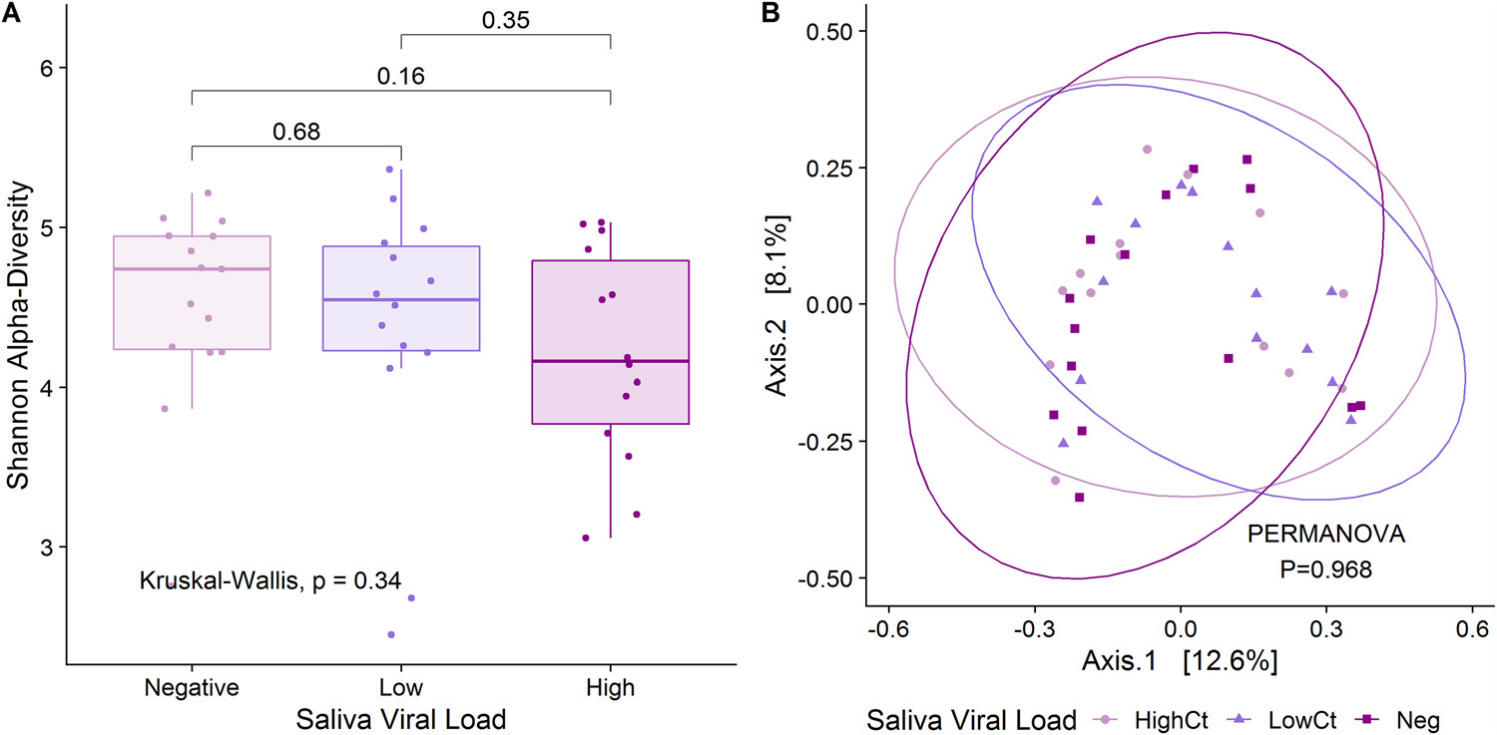
Saliva microbiome diversity compared across SARS-CoV-2 saliva viral load. (A) Shannon α-diversity and (B) UniFrac β-diversity in saliva collected from patients with SARS-CoV-2-positive nasopharyngeal swabs via clinical testing upon admission. Saliva samples were stratified by SARS-CoV-2 RT-PCR cycle threshold (*C_T_*) values into the following categories: negative (*C_T_* > 40), low viral load (*C_T_* > 30), and high viral load (*C_T_* < 30). Kruskal-Wallis test was used to compare Shannon α-diversity, and permutational ANOVA (PERMANOVA) was used to compare UniFrac β-diversity. Normal data ellipses for each group are shown over the β-diversity plot in panel B.

**FIG 3:**
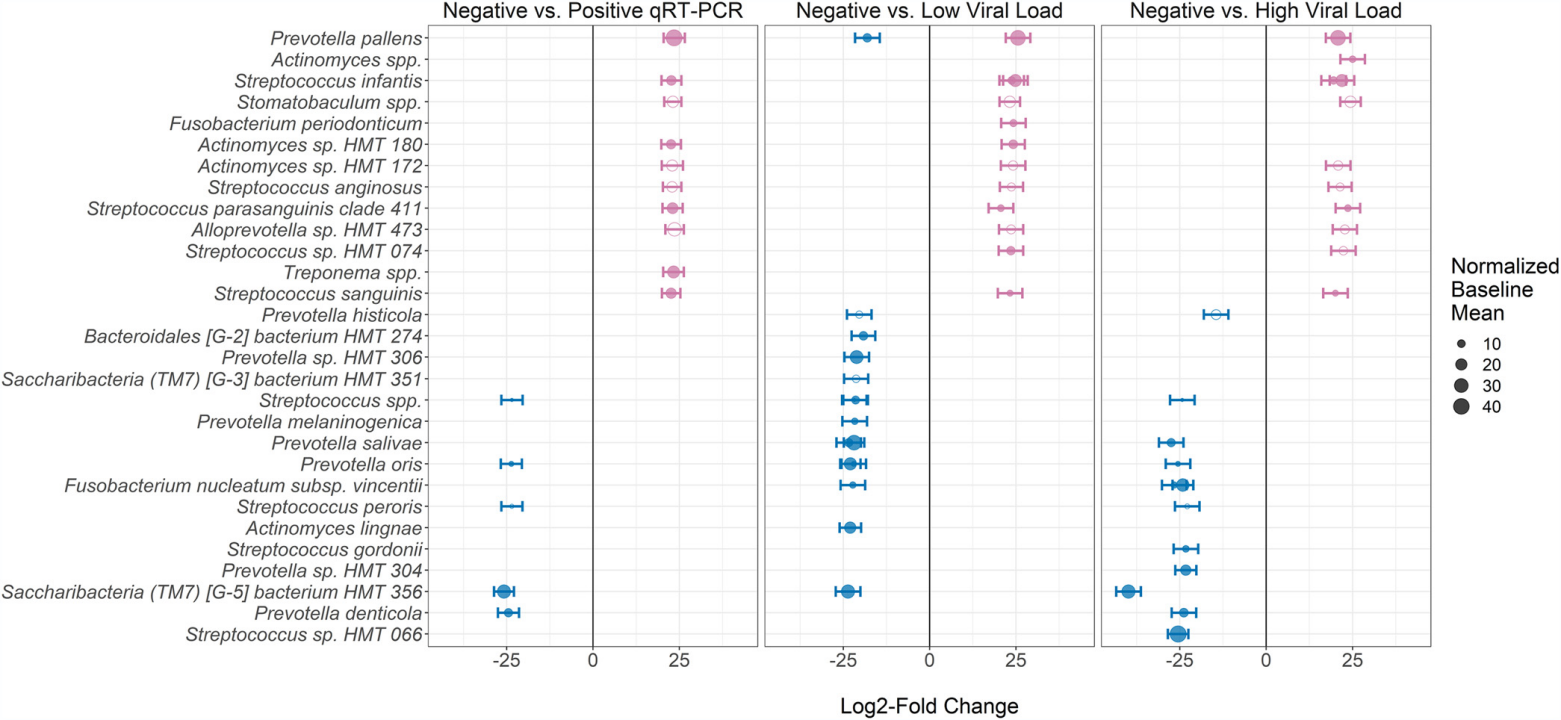
Differentially abundant taxa based on SARS-CoV-2 viral load in saliva from SARS-CoV-2-positive patients. Panels show taxa classified at the genus, species, and/or strain level, which are differentially enriched (*DESeq2*, Benjamini-Hochberg *P*_adj_ < 0.05) across the following comparisons (left to right): (i) SARS-CoV-2-positive (pink) versus -negative (blue) saliva, (ii) SARS-CoV-2-positive with low viral load (pink) versus negative (blue) saliva, and (iii) SARS-CoV-2-positive with high viral load (pink) versus negative (blue) saliva. Median cycle threshold (*C_T_*) across all positive samples was used to determine the threshold between low (*C_T_* > 30) versus high (*C_T_* < 30) viral load. Each point represents one amplicon sequence variant (ASV), scaled in size by the average normalized read counts in baseline (saliva SARS-CoV-2 negative) samples (i.e., larger circles represent ASVs with higher read counts in baseline samples). Filled circles represent ASVs which remained differentially abundant after adjusting for whether the patient received supplemental oxygen to account for effects of oxygen on the microbiome. Standard error bars for the log_2_ fold change calculation across groups are shown for each point. Note: for low-viral-load versus negative saliva samples, different *P. pallens* ASVs were enriched in either group (middle panel, first row). For all other species and strains, all ASVs assigned to a specific taxon were differentially enriched in the same group.

We next tested whether SARS-CoV-2 viral load in saliva was associated with saliva microbiome diversity or composition. There were no differences in Shannon (*P* = 0.34) or Chao (*P* = 0.66) α-diversity based on saliva viral load, and there was no evidence of clustering by viral load on β-diversity analyses (PERMANOVA *P* = 0.97) (Fig. 2; Fig. S1). However, we identified multiple taxa that were differentially abundant between saliva-negative COVID-19 patients and those with detectable saliva viral load, with alterations in numerous ASVs in genera Streptococcus, *Prevotella*, and *Actinomyces*, among others (Fig. 3; Table S4). COVID-19 patients with detectable SARS-CoV-2 in saliva had increased P. pallens, Streptococcus infantis, Streptococcus parasanguinis clade 411, Streptococcus sanguinis, *Actinomyces* sp. HMT180, and Treponema spp. and decreased Prevotella denticola, Prevotella oris, *Saccharibacteria* strain HMT356, Streptococcus peroris, and an additional Streptococcus spp. Comparisons of COVID-19 patients with low viral load versus undetectable virus and high viral load versus undetectable virus produced highly similar results (Fig. 3; Table S4).

While there was no difference in the proportion of COVID-19 patients requiring supplemental oxygen based on saliva viral load (Table S3), we speculated that receipt of supplemental oxygen could affect the relationship between saliva viral load and microbiome composition. However, after adjusting the DESeq2 generalized linear model for receipt of supplemental oxygen, the large majority of originally identified taxa that were altered based on saliva viral load remained significantly differentially abundant (Fig. 3; Table S4).

## DISCUSSION

In our cohort of hospitalized patients, we did not find marked differences in the saliva microbiome between those with and without COVID-19 disease. However, among COVID-19 patients, we did find significant differences in saliva microbiome communities based on saliva SARS-CoV-2 viral load. Further, differential abundance of these taxa was also independent of administration of supplemental oxygen, which suggests that the addition of oxygen into the local oral environment by either nasal cannula or face mask does not markedly affect the relationship between the microbiome and viral load.

Prior studies of other respiratory viruses, such as influenza, have established that the saliva microbiome can be a mediator of disease severity. Bacterial-viral interactions between neuraminidase-producing streptococci and influenza may directly affect viral load (9). The nasal and posterior pharyngeal microbiome is highly distinct in household contacts who do or do not develop influenza after close contact exposure to an index patient (11), which suggests that host microbiome influences susceptibility to influenza infection after exposure. Recently, evidence has been emerging that similar transkingdom relationships may exist with SARS-CoV-2. Analyses of bronchoalveolar lavage fluid from COVID-19 patients and controls identified alterations in bacterial communities capable of modifying heparan sulfate, which is required for SARS-CoV-2 binding to angiotensin converting enzyme 2 (ACE2) (8). Bacterial components of both the respiratory and gut microbiomes influence expression of and cofactors needed for viral binding to ACE2, the host-derived receptor for SARS-CoV-2 (8, 12, 13). Our findings are also consistent with bacterial-viral associations which may have potential clinical significance and warrant further study. Additionally, the role of inflammation in the oral microbiome should also be considered. One hallmark of COVID-19 disease is a robust inflammatory response in patients, which can lead to a hyperinflammatory state and death (8, 14–16). Specific components of the oral microbiome, such as Prevotella nigrescens and Streptococcus mutans, are known to be associated with inflammation and oral dysbiosis (3). It is also possible that COVID-19-induced inflammation could directly alter oral bacterial composition. Further investigation into the effect of inflammation on the oral microbiome during SARS-CoV-2 infection is necessary.

Interestingly, we observed only minimal differences in the saliva microbiome of COVID-19 patients compared to that of controls. This suggests that the presence of SARS-CoV-2 may not in and of itself markedly alter the oral microbiome. This is consistent with previous work demonstrating that influenza infection does not appreciably affect oropharyngeal microbiome composition (17). However, possible mediation by the microbiome of susceptibility to SARS-CoV-2 infection cannot be excluded, as we did not assess SARS-CoV-2 exposure in controls. However, two other studies did recently observe an oral microbiome diversity in COVID-19 patients significantly lower than that in healthy controls (18, 19), along with a decrease in butyrate-producing bacteria (18). A metagenomic analysis identified enrichment in opportunistic oral pathogens *Veillonella* and *Megasphaera* in COVID-19 patients, but, similarly to this study, did not find significant changes in alpha-diversity when comparing noncritically ill COVID-19 patients to healthy controls (20). These findings taken along with the findings in this study underscore the need for larger studies of the oral microbiome in COVID-19 patients.

Assessing the diagnostic performance of saliva testing for COVID-19 was not a focus of the current study, and the study was underpowered to identify clinical features associated with SARS-CoV-2 viral load in saliva. However, the results do add to growing data on saliva testing to identify COVID-19 patients. In this study, RT-PCR for SARS-CoV-2 in the saliva had lower sensitivity compared to that seen in prior reports on saliva-based testing in patients hospitalized with COVID-19 disease (1, 21, 22). These studies included patients presenting with more severe disease, and saliva testing may have decreased sensitivity in less symptomatic and asymptomatic patients. Another consideration is the quality of saliva sample the patients produce. COVID-19 patients can have difficulty producing saliva (23, 24), and hyposalivation in COVID-19 patients can be exacerbated by delivery of supplementary oxygen, especially via facemask. Both hyposalivation and supplemental oxygen delivery could affect the production of high-quality saliva samples and adversely affect the performance of RT-PCR SARS-CoV-2 testing on these samples in real-life clinical situations.

This study represents one of the first comprehensive analyses of the saliva microbiome in patients with active, symptomatic SARS-CoV-2 infection. Strengths include the rapid saliva collection in newly admitted patients, the availability of relevant clinical and laboratory data for both cases and controls, and the ability to integrate RT-PCR and 16S rRNA sequencing data on the saliva samples collected. The study included a large proportion of black and Hispanic patients, populations disproportionately affected by COVID-19 yet historically underrepresented in clinical studies. Limitations include a relatively small sample size, which may have affected our ability to detect biologically meaningful differences in microbiome composition between COVID-19 patients and controls. Given the extraordinary circumstances the medical center faced at the height of the COVID-19 surge in New York City, recruiting and consenting patients for research studies was challenging. Clinical *C_T_* values for corresponding nasopharyngeal specimens from this cohort were also largely unavailable due to these constraints. Assessment of the corresponding nasopharyngeal microbiome may have been informative but was beyond the scope of this study. Additionally, data on oral health such as periodontal disease were not available, so any relationship between oral health and the oral microbiome could not be evaluated in this study. Future studies are needed to address whether oral health and preexisting periodontal disease affect the saliva microbiome during SARS-CoV-2 infection.

As the COVID-19 pandemic continues to pose a global public health threat, it is essential to understand how the virus interacts with the host and how these interactions affect severity of disease. The saliva microbiome represents an array of commensal and potentially pathogenic bacteria which can act as cofactors in the disease process. With high rates of hospitalized patients with COVID-19, common complications, such as bacterial pneumonia, underscore the importance of understanding viral-bacterial community dynamics. Further studies are needed to better elucidate the mechanisms of interactions between the saliva microbiome and SARS-CoV-2 and what effect these interactions have on the course of COVID-19 disease. Additionally, an improved understanding of saliva microbiome dynamics in the setting of SARS-CoV-2 infection may allow for the development of strategies to decrease transmission and complications.

## MATERIALS AND METHODS

### Sample processing and nucleic acid extraction.

Consented patients provided approximately 1 to 2 ml saliva via self-collection in Zymo DNA/RNA Shield saliva collection kits containing 2 ml Zymo DNA/RNA Shield for viral inactivation and stabilization. Inactivated samples were separated into two 1-ml aliquots and stored at −80°C. DNA and RNA were extracted in parallel from 300 μl sample using the ZymoBIOMICS DNA/RNA miniprep kit (Zymo).

### SARS-CoV-2 RT-PCR.

We used the 2019-nCoV research use only (RUO) primers and probes kit to amplify the SARS-CoV-2 N1 gene and human RNAse P (RP) gene (IDT). Reverse transcription PCR (RT-PCR) was performed using the TaqMan Fast Virus 1-step master mix (ThermoFisher), with a total reaction volume of 20 μl, including 5 μl of RNA template. Single RT-PCRs were performed for each sample on 96-well plates, with each plate including a no template control (NTC) with 5 μl of molecular-grade water. Cycle threshold (*C_T_*) values were obtained from RT-PCR amplification curves, and samples with *C_T_* of <40 were considered SARS-CoV-2 positive.

### 16S rRNA sequencing and data processing.

We amplified the 16S rRNA V1-V2 region using Illumina adapter-ligated primers (25), with 2.5 μl (5 ng) DNA template in a total reaction volume of 25 μl (12.5 μl KAPA HiFi HotStart ReadyMix, 5 μl each of forward and reverse primers) with the following cycling protocol: 95°C for 3 min, 25 cycles of 95°C for 30s, 55°C for 30s, and 72°C for 30s, and 72°C for 5 min. The Illumina Nextera XT v2 index sets A to D were used to barcode sequencing libraries. Libraries were sequenced on an Illumina MiSeq using the v3 reagent kit (600 cycles) and a loading concentration of 12 pM with 10% phiX spike-in.

Raw sequencing reads were adapter-trimmed and demultiplexed after FASTQ conversion in BaseSpace (Illumina). Quantitative Insights Into Microbial Ecology (QIIME2) and DADA2 were used to trim, dereplicate, and filter chimeric sequences before generating amplicon sequence variant (ASV) tables (26). Based on the quality score profiles of sequencing reads, forward reads were truncated at 280 bp and reverse reads were truncated at 260 bp prior to merging, ambiguities in the overlap region were not allowed, and default parameters were otherwise applied in the R *dada2* package filterAndTrim() function [truncLen=c(280,260); trimLeft=c(5,5), maxN = 0, maxEE=c(2,2), truncQ = 2]. After dereplication and merging of reads, chimeric reads were identified by consensus across samples using the *dada2* function removeBimeraDenovo(). All samples passed the imposed minimum of 10,000 reads after quality filtering for inclusion in this analysis. The MAFFT and FastTree modules in QIIME2 were used to generate a phylogenetic tree of all ASV sequences. ASV taxonomy was assigned using a naive Bayesian classifier trained on a custom database consisting of Greengenes 99% clustered operational taxonomic units (OTUs) and the eHOMD 16S rRNA RefSeq version 15.2 database for increased resolution of oral taxa assignments (27, 28). Unique sequences across the Greengenes and eHOMD databases were clustered at 99% identity and used as the training data set for the QIIME2 naive Bayesian taxonomic classifier. Read counts before and after quality filtering and ASV assignment are provided in Table S1. The R *phyloseq* package was used to calculate microbiome α- and β-diversity metrics (Shannon, Chao indices, and UniFrac distance matrix) (29).

### Quantification and statistical analysis.

Categorical variables were compared using chi-square or Fisher’s exact test. Continuous variables were assessed for normal or normal-like distribution using visualization of QQ-plots. Variables with normal or near-normal distribution were compared using two-sided *t*-tests, and nonnormally distributed variables were compared using Kruskal-Wallis or Mann-Whitney-U nonparametric tests. UniFrac β-diversity distance matrices were compared across groups using permutation ANOVA (PERMANOVA). For all comparisons, significance was defined as a *P* value of <0.05. Differential abundance analyses were performed using DESeq2, with significance defined as Benjamini-Hochberg adjusted *P*_adj_ of <0.05. The statistical tests used are clearly indicated for each comparison throughout the text and in table and figure legends. All statistical analysis and table and figure generation were performed using R version 3.6.1, and R Markdown files detailing all statistical analyses and visualizations are available in a public GitHub repository (https://github.com/mka2136/covid19_saliva_microbiome).

### Experimental model and subject details.

This was a prospective cohort study of patients newly admitted to the medical service at Columbia University Irving Medical Center between 7 April 2020 and 9 May 2020. The study was approved by the Columbia University Institutional Review Board. The electronic medical record was screened to identify patients admitted within the previous 24 h to an inpatient, non-intensive care medical service. Inclusion criteria included age of ≥18 years, ability to produce a saliva sample, and ability to provide informed consent. Patients were excluded if they were intubated, otherwise medically unstable or unconscious, or admitted to an intensive care setting. Hospitalized patients with a nasopharyngeal swab negative for SARS-CoV-2 but with history of prior COVID-19 disease were excluded. Patients included in the control group had a negative nasopharyngeal swab for SARS-CoV-2 on admission, as per our institution’s clinical practice at the time of the study, and otherwise met the same inclusion and exclusion criteria as those in the case group.

Per routine clinical practice during the study period, all patients were tested in the emergency room for SARS-CoV-2 by nasopharyngeal swab PCR. Written informed consent was obtained from SARS-CoV-2-negative patients. Verbal consent was obtained from patients with a positive or pending SARS-CoV-2 test to minimize patient contact.

The following patient-level data were extracted from the electronic medical record: age, sex, race, ethnicity, body mass index (BMI), comorbidities, and receipt of antibiotics prior to or at admission. Additional hospitalization data collected included oxygen requirement at the time of admission, COVID-19 treatment (including methylprednisolone and remdesivir) received during the hospitalization, and relevant laboratory results (white blood cells [WBC], C-reactive protein [CRP], erythrocyte sedimentation rate [ESR], neutrophil-to-lymphocyte ratio [NLR], interleukin-6 [IL-6], ferritin). Clinical outcomes were recorded, including hospital length of stay, clinical decompensation (defined as need for intubation and/or transfer to an intensive care setting), discharge to hospice, discharge to home, and death. All details regarding sample size and demographic characteristics of the full cohort are included in Table 1.

The study was approved by the Columbia University Institutional Review Board (protocol number IRB-AAAS9837).

### Data availability.

Raw sequencing reads generated in this study have been deposited to the NCBI Short Read Archive (SRA) under NCBI BioProject PRJNA669421. All R code used for statistical analyses and table and figure generation are available in a public GitHub repository (https://github.com/mka2136/saliva_covid19_microbiome). Further information and requests for resources or reagents should be directed to the lead contact, Julian A. Abrams. This study did not generate new unique reagents.

## References

[1] Azzi L, Carcano G, Gianfagna F, Grossi P, Gasperina DD, Genoni A, Fasano M, Sessa F, Tettamanti L, Carinci F, Maurino V, Rossi A, Tagliabue A, Baj A. 2020. Saliva is a reliable tool to detect SARS-CoV-2. J Infect 81:e45–e50. doi:10.1016/j.jinf.2020.04.005. 32298676 PMC7194805

[2] Han P, Ivanovski S. 2020. Saliva—friend and foe in the COVID-19 outbreak. Diagnostics 10:290. doi:10.3390/diagnostics10050290. 32397487 PMC7277967

[3] Kleinstein SE, Nelson KE, Freire M. 2020. Inflammatory networks linking oral microbiome with systemic health and disease. J Dent Res 99:1131–1139. doi:10.1177/0022034520926126. 32459164 PMC7443998

[4] Xiang Z, Koo H, Chen Q, Zhou X, Liu Y, Simon-Soro A. 2020. Potential implications of SARS-CoV-2 oral infection in the host microbiota. J Oral Microbiol 13:1853451. doi:10.1080/20002297.2020.1853451. 33312449 PMC7711743

[5] Williams DW, Greenwell-Wild T, Brenchley L, Dutzan N, Overmiller A, Sawaya AP, Webb S, Martin D, Hajishengallis G, Divaris K, Morasso M, Haniffa M, Moutsopoulos NM, NIDCD/NIDCR Genomics and Computational Biology Core. 2021. Human oral mucosa cell atlas reveals a stromal-neutrophil axis regulating tissue immunity. Cell 184:4090–4104. doi:10.1016/j.cell.2021.05.013. 34129837 PMC8359928

[6] Patel J, Sampson V. 2020. The role of oral bacteria in COVID-19. Lancet Microbe 1:e105. doi:10.1016/S2666-5247(20)30057-4. 32835339 PMC7333982

[7] Honarmand Ebrahimi K. 2020. SARS‐CoV‐2 spike glycoprotein‐binding proteins expressed by upper respiratory tract bacteria may prevent severe viral infection. FEBS Lett 594:1651–1660. doi:10.1002/1873-3468.13845. 32449939 PMC7280584

[8] Martino C, Kellman BP, Sandoval DR, Clausen TM, Marotz CA, Song SJ. 2020. Bacterial modification of the host glycosaminoglycan heparan sulfate modulates SARS-CoV-2 infectivity. bioRxiv. 10.1101/2020.08.17.238444.

[9] Kamio N, Imai K, Shimizu K, Cueno ME, Tamura M, Saito Y, Ochiai K. 2015. Neuraminidase-producing oral mitis group streptococci potentially contribute to influenza viral infection and reduction in antiviral efficacy of zanamivir. Cell Mol Life Sci 72:357–366. doi:10.1007/s00018-014-1669-1. 25001578 PMC11113501

[10] Magleby R, Westblade LF, Trzebucki A, Simon MS, Rajan M, Park J, Goyal P, Safford MM, Satlin MJ. 2020. Impact of SARS-CoV-2 viral load on risk of intubation and mortality among hospitalized patients with coronavirus disease 2019. Clin Infect Dis. doi:10.1093/cid/ciaa851. PMC7337625 32603425

[11] Tsang TK, Lee KH, Foxman B, Balmaseda A, Gresh L, Sanchez N, Ojeda S, Lopez R, Yang Y, Kuan G, Gordon A. 2020. Association between the respiratory microbiome and susceptibility to influenza virus infection. Clin Infect Dis 71:1195–1203. doi:10.1093/cid/ciz968. 31562814 PMC7442850

[12] Hashimoto T, Perlot T, Rehman A, Trichereau J, Ishiguro H, Paolino M, Sigl V, Hanada T, Hanada R, Lipinski S, Wild B, Camargo SMR, Singer D, Richter A, Kuba K, Fukamizu A, Schreiber S, Clevers H, Verrey F, Rosenstiel P, Penninger JM. 2012. ACE2 links amino acid malnutrition to microbial ecology and intestinal inflammation. Nature 487:477–481. doi:10.1038/nature11228. 22837003 PMC7095315

[13] Yang T, Chakraborty S, Saha P, Mell B, Cheng X, Yeo J-Y, Mei X, Zhou G, Mandal J, Golonka R, Yeoh BS, Putluri V, Piyarathna DWB, Putluri N, McCarthy CG, Wenceslau CF, Sreekumar A, Gewirtz AT, Vijay-Kumar M, Joe B. 2020. Gnotobiotic rats reveal that gut microbiota regulates colonic mRNA of Ace2, the receptor for SARS-CoV-2 infectivity. Hypertension 76:e1–e3. doi:10.1161/HYPERTENSIONAHA.120.15360. 32426999 PMC7379164

[14] Cummings MJ, Baldwin MR, Abrams D, Jacobson SD, Meyer BJ, Balough EM, Aaron JG, Claassen J, Rabbani LE, Hastie J, Hochman BR, Salazar-Schicchi J, Yip NH, Brodie D, O’Donnell MR. 2020. Epidemiology, clinical course, and outcomes of critically ill adults with COVID-19 in New York City: a prospective cohort study. Lancet 395:1763–1770. doi:10.1016/S0140-6736(20)31189-2. 32442528 PMC7237188

[15] Wu Z, McGoogan JM. 2020. Characteristics of and important lessons from the coronavirus disease 2019 (COVID-19) outbreak in China. JAMA 323:1239–1242. doi:10.1001/jama.2020.2648. 32091533

[16] Huang C, Wang Y, Li X, Ren L, Zhao J, Hu Y, Zhang L, Fan G, Xu J, Gu X, Cheng Z, Yu T, Xia J, Wei Y, Wu W, Xie X, Yin W, Li H, Liu M, Xiao Y, Gao H, Guo L, Xie J, Wang G, Jiang R, Gao Z, Jin Q, Wang J, Cao B. 2020. Clinical features of patients infected with 2019 novel coronavirus in Wuhan, China. Lancet 395:497–506. doi:10.1016/S0140-6736(20)30183-5. 31986264 PMC7159299

[17] Ramos-Sevillano E, Wade WG, Mann A, Gilbert A, Lambkin-Williams R, Killingley B, Nguyen-Van-Tam JS, Tang CM. 2019. The effect of influenza virus on the human oropharyngeal microbiome. Clin Infect Dis 68:1993–2002. doi:10.1093/cid/ciy821. 30445563 PMC6541733

[18] Ren Z, Wang H, Cui G, Lu H, Wang L, Luo H, Chen X, Ren H, Sun R, Liu W, Liu X, Liu C, Li A, Wang X, Rao B, Yuan C, Zhang H, Sun J, Chen X, Li B, Hu C, Wu Z, Yu Z, Kan Q, Li L. 2021. Alterations in the human oral and gut microbiomes and lipidomics in COVID-19. Gut 70:1253–1265. doi:10.1136/gutjnl-2020-323826. 33789966 PMC8042598

[19] Iebba V, Zanotta N, Campisciano G, Zerbato V, Di Bella S, Cason C, Luzzati R, Confalonieri M, Plamara AT, Comar M. 2020. Profiling of oral microbiota and cytokines in COVID-19 patients. Front Microbiol 12:671813. doi:10.1101/2020.12.13.422589. PMC8361794 34394024

[20] Ma S, Zhang F, Zhou F, Li H, Ge W, Gan R, Nie H, Li B, Wang Y, Wu M, Li D, Wang D, Wang Z, You Y, Huang Z. 2021. Metagenomic analysis reveals oropharyngeal microbiota alterations in patients with COVID-19. Signal Transduct Target Ther 6:191. doi:10.1038/s41392-021-00614-3. 33986253 PMC8116522

[21] To KK-W, Tsang OT-Y, Yip CC-Y, Chan K-H, Wu T-C, Chan JM-C, Leung W-S, Chik TS-H, Choi CY-C, Kandamby DH, Lung DC, Tam AR, Poon RW-S, Fung AY-F, Hung IF-N, Cheng VC-C, Chan JF-W, Yuen K-Y. 2020. Consistent detection of 2019 novel coronavirus in saliva. Clin Infect Dis 71:841–843. doi:10.1093/cid/ciaa149. 32047895 PMC7108139

[22] Wyllie AL, Fournier J, Casanovas-Massana A, Campbell M, Tokuyama M, Vijayakumar P, Warren JL, Geng B, Muenker MC, Moore AJ, Vogels CBF, Petrone ME, Ott IM, Lu P, Venkataraman A, Lu-Culligan A, Klein J, Earnest R, Simonov M, Datta R, Handoko R, Naushad N, Sewanan LR, Valdez J, White EB, Lapidus S, Kalinich CC, Jiang X, Kim DJ, Kudo E, Linehan M, Mao T, Moriyama M, Oh JE, Park A, Silva J, Song E, Takahashi T, Taura M, Weizman O-E, Wong P, Yang Y, Bermejo S, Odio CD, Omer SB, Dela Cruz CS, Farhadian S, Martinello RA, Iwasaki A, Grubaugh ND, et al. 2020. Saliva or nasopharyngeal swab specimens for detection of SARS-CoV-2. N Engl J Med 383:1283–1286. doi:10.1056/NEJMc2016359. 32857487 PMC7484747

[23] Baghizadeh Fini M. 2020. Oral saliva and COVID-19. Oral Oncol 108:104821. doi:10.1016/j.oraloncology.2020.104821. 32474389 PMC7250788

[24] Freni F, Meduri A, Gazia F, Nicastro V, Galletti C, Aragona P, Galletti C, Galletti B, Galletti F. 2020. Symptomatology in head and neck district in coronavirus disease (COVID-19): a possible neuroinvasive action of SARS-CoV-2. Am J Otolaryngol 41:102612. doi:10.1016/j.amjoto.2020.102612. 32574896 PMC7301823

[25] Schirmer M, Ijaz UZ, D’Amore R, Hall N, Sloan WT, Quince C. 2015. Insight into biases and sequencing errors for amplicon sequencing with the Illumina MiSeq platform. Nucleic Acids Res 43:e37. doi:10.1093/nar/gku1341. 25586220 PMC4381044

[26] Bolyen E, Rideout JR, Dillon MR, Bokulich NA, Abnet CC, Al-Ghalith GA, Alexander H, Alm EJ, Arumugam M, Asnicar F, Bai Y, Bisanz JE, Bittinger K, Brejnrod A, Brislawn CJ, Brown CT, Callahan BJ, Caraballo-Rodríguez AM, Chase J, Cope EK, Da Silva R, Diener C, Dorrestein PC, Douglas GM, Durall DM, Duvallet C, Edwardson CF, Ernst M, Estaki M, Fouquier J, Gauglitz JM, Gibbons SM, Gibson DL, Gonzalez A, Gorlick K, Guo J, Hillmann B, Holmes S, Holste H, Huttenhower C, Huttley GA, Janssen S, Jarmusch AK, Jiang L, Kaehler BD, Kang KB, Keefe CR, Keim P, Kelley ST, Knights D, et al. 2019. Reproducible, interactive, scalable and extensible microbiome data science using QIIME 2. Nat Biotechnol 37:852–857. doi:10.1038/s41587-019-0209-9. 31341288 PMC7015180

[27] Escapa IF, Chen T, Huang Y, Gajare P, Dewhirst FE, Lemon KP. 2018. New insights into human nostril microbiome from the expanded human oral microbiome database (eHOMD): a resource for the microbiome of the human aerodigestive tract. mSystems 3:e00187-18. doi:10.1128/mSystems.00187-18. PMC6280432 30534599

[28] F Escapa I, Huang Y, Chen T, Lin M, Kokaras A, Dewhirst FE, Lemon KP. 2020. Construction of habitat-specific training sets to achieve species-level assignment in 16S rRNA gene datasets. Microbiome 8:65. doi:10.1186/s40168-020-00841-w. 32414415 PMC7291764

[29] McMurdie PJ, Holmes S. 2013. phyloseq: an R package for reproducible interactive analysis and graphics of microbiome census data. PLoS One 8:e61217. doi:10.1371/journal.pone.0061217. 23630581 PMC3632530

