# Supplementary material for: Oral Microbiome Alterations and SARS-CoV-2 Saliva Viral Load in Patients with COVID-19
Source: Microbiol Spectr. 2021 Oct 13;9(2):e00055-21. doi: 10.1128/Spectrum.00055-21 (PMC8515944; doi:10.1128/Spectrum.00055-21)
Supplement: Supplemental file 1 — Supplemental material. Download SPECTRUM00055-21_Supp_1_seq7.pdf, PDF file, 0.2 MB [file spectrum00055-21_supp_1_seq7.pdf]

Supplemental Information

Table S1. Diagnoses of COVID-19 negative patients

Patient ID	Age	Sex	Primary Diagnosis	Secondary Diagnosis
**2**	53	Male	Syncope	Metabolic acidosis from missing dialysis
**3**	34	Female	Vaginal bleeding	Retained products of conception
**7**	32	Male	Hypoxia	Community acquired pneumonia
**9**	80	Female	Poor oral Intake	Non-ST elevation myocardial infarction
**12**	65	Female	Pneumonia	
**13**	76	Female	Altered mental status	MSSA bacteremia
**16**	71	Male	Dizziness	Hypovolemia
**18**	41	Male	Weakness	Diabetic ketoacidosis
**22**	53	Male	GI bleed	
**23**	64	Female	Shortness of breath	CHF exacerbation
**26**	68	Female	Hypoxia	Pulmonary embolism
**27**	23	Male	Drainage from axillary wound	Anemia
**31**	52	Female	Placement of TDC for initiation of dialysis	
**32**	52	Female	Rectal bleeding	Sickle cell pain crisis
**34**	88	Male	Shortness of breath	CHF exacerbation
**37**	57	Male	Chest pain	Acute coronary syndrome
**39**	22	Female	Nausea/vomiting	Diabetic ketoacidosis
**41**	64	Male	Diarrhea	
**49**	85	Female	Pleuritic chest pain	CHF exacerbation
**52**	49	Male	Shortness of breath	CHF exacerbation
**56**	55	Female	Chest pain	
**57**	30	Female	Abdominal pain	Acute pancreatitis
**60**	68	Male	Dizziness	Hyperglycemia
**61**	67	Male	Pleural effusion	
**62**	66	Male	Chest pain	Non-ST elevation myocardial infarction
**64**	62	Male	Chest pain	CHF exacerbation
**70**	32	Male	Cough	Acute kidney injury
**71**	40	Male	Chest pain	Non-ST elevation myocardial infarction
**72**	83	Female	Fall	
**73**	66	Male	Sternal wound infection	
**74**	55	Male	Fall	L1 fracture
**75**	53	Male	Fatigue	Hypercalcemia from PTLD
**76**	22	Male	Shortness of breath	Asthma exacerbation
**80**	62	Male	Fatigue	Acute kidney injury
**81**	26	Female	Fatigue	Diabetic ketoacidosis

**82**	56	Female	Shortness of breath	COPD exacerbation
**84**	44	Female	Urosepsis	
**85**	71	Female	Abdominal pain	
**87**	51	Female	Fatigue	
**90**	51	Male	Cough	Pulmonary embolism
**95**	58	Male	Facial swelling	
**96**	72	Male	Weakness	Transient hypotension
**97**	35	Male	Lower extremity cellulitis	
**98**	30	Male	Sickle cell pain crisis	
**99**	65	Male	Cough	
**100**	77	Female	Syncope	Orthostatic hypotension
**103**	59	Male	Lower extremity pain	Deep vein thrombosis
**104**	75	Female	Failure to thrive	Urinary tract infection
**105**	64	Female	Palpitations	Non-ST elevation myocardial infarction
**106**	39	Male	Abdominal pain	HLH
**107**	71	Male	Weakness	
**108**	76	Male	Shortness of breath	CHF exacerbation
**110**	61	Female	Chronic hypoxia	Obstructive sleep apnea
**111**	43	Female	Fatigue	Anemia
**113**	54	Male	Cough	
**114**	47	Male	Cancer related pain	
**115**	67	Male	Back pain	Metastatic prostate cancer
**116**	76	Male	Shortness of breath	CHF exacerbation
**118**	56	Male	Transaminitis	

Abbreviations: MSSA – methicillin-sensitive *Staphylococcus aureus*; TDC – tunneled dialysis catheter; CHF – congestive heart failure; PTLD – post-transplant lymphoproliferative disorder; COPD – chronic obstructive pulmonary disease; HLH – hemophagocytic lymphohistiocytosis

Table S2. Demographic and clinical characteristics of COVID-19 patients stratified by saliva SARS-CoV-2 viral load.

	Negative [1] (n=15)	Low viral load [1] (n=14)	High viral load [1] (n=14)	p [2]
Saliva SARS-CoV-2 Ct (median [IQR]) [1]	-	34.7 [33.5-35.6]	24.8 [20.6-26.6]	
Age (mean (SD))	56.5 (17.6)	56.4 (15.6)	49.7 (15.9)	0.46
Male (%)	6 (40.0)	6 (42.9)	6 (42.9)	0.98
Race (%)				0.63
Black	7 (46.7)	3 (21.4)	6 (42.9)	
White	3 (20.0)	3 (21.4)	3 (21.4)	
Other/unreported	5 (33.3)	8 (57.1)	5 (35.7)	
Ethnicity (%)				0.07
Hispanic/Latinx	3 (20.0)	9 (64.3)	7 (50.0)	
Not Hispanic/Latinx	10 (66.7)	2 (14.3)	5 (35.7)	
Not specified	2 (13.3)	3 (21.4)	2 (14.3)	
BMI (median [IQR])	30.2 [25.0, 34.3]	27.3 [21.2, 37.0]	31.0 [27.2, 35.9]	0.37 [3]
HTN (%)	7 (46.7)	8 (57.1)	7 (50.0)	0.85
DM (%)	5 (33.3)	5 (35.7)	6 (42.9)	0.86
Underlying kidney disease (%)	1 (6.7)	4 (28.6)	1 (7.1)	0.16
Any pulmonary disease (%)	4 (26.7)	3 (21.4)	2 (14.3)	0.71
Charlson Comorbidity Index (median [IQR])	2.0 [0.5, 4.0]	2.0 [1.0, 4.0]	2.0 [0.0, 3.0]	0.59 [3]
Oxygen rank severity (%)				0.32 [4]
No supplemental oxygen	12 (80.0)	9 (64.3)	7 (50.0)	
Nasal cannula	3 (20.0)	3 (21.4)	6 (42.9)	
Non-invasive ventilation	0 (0.0)	2 (14.3)	1 (7.1)	
IL-6 (median [IQR]) [5]	10.4 [6.3, 25.9]	25.2 [20.4, 38.6]	17.9 [11.7, 24.2]	0.25 [3]
ESR (median [IQR]) [5]	56.5 [48.5, 109]	60.0 [50.0, 87.5]	67.0 [57.0, 83.0]	0.94 [3]
CRP (median [IQR]) [5]	63.8 [14.4, 129]	35.0 [17.2, 113]	104 [64.8, 176]	0.17 [3]
Ferritin (median [IQR]) [5]	560 [213, 911]	343 [262, 647]	567 [378, 1945]	0.45 [3]
D-dimer (median [IQR]) [5]	0.99 [0.72, 6.93]	0.92 [0.76, 1.31]	0.78 [0.53, 2.07]	0.61 [3]
WBC (median [IQR]) [5]	7.30 [5.75, 8.78]	6.91 [5.50, 9.11]	6.65 [4.61, 11.5]	0.93 [3]
NLR (median [IQR]) [5]	3.68 [3.19, 5.01]	3.67 [2.85, 6.16]	4.75 [2.19, 15.8]	0.78 [3]
Symptom duration >3 d (%) [6]	5 (33.3)	5 (35.7)	4 (28.6)	1.00 [4]
Antibiotics ≤48 hrs prior to saliva collection (%) [7]	7 (46.7)	6 (42.9)	8 (57.1)	0.74
Composite outcome (%)				0.54 [4]
Deceased	1 (6.7)	1 (7.1)	1 (7.1)	
Decompensated (no death)	0 (0.0)	0 (0.0)	2 (14.3)	
Discharged (no death)	14 (93.3)	13 (92.9)	11 (78.6)	

[1] As determined via testing of saliva using qRT-PCR. Cycle threshold (Ct) values below 40 were considered positive, and median Ct value was used to stratify low (Ct>30) versus high (Ct<30) viral load
[2] Categorical variables were compared using the Chi-squared test, unless indicated as below. Continuous variables were assessed for normal or normal-like distribution and compared using t-tests, unless indicated as below.

[3] Kruskal-Wallis test was used due to non-normal distribution
[4] Fisher's exact test was used due to at least one expected value ≤ 5
[5] Lab values as measured upon admission; not available for all patients (IL-6 n=30/43; ESR n=38/43; CRP n=41/43; Ferritin n=41/43; D-dimer n=37/43; WBC n=43/43; NLR n=39/43)
[6] Reported duration of symptoms prior to hospitalization
[7] Inpatient antibiotic use, determined through chart review

Abbreviations: BMI – body mass index; HTN – hypertension; DM – diabetes mellitus; CAD – coronary artery disease; ESR – erythrocyte sedimentation rate; CRP – C-reactive protein; WBC – white blood cell count; NLR – neutrophil-lymphocyte ratio
